# Genetic insights: High germline variant rate in an indigenous African cohort with early-onset colorectal cancer

**DOI:** 10.3389/fonc.2023.1253867

**Published:** 2023-10-27

**Authors:** Safiye Yildiz, Takudzwa N. Musarurwa, Ursula Algar, Ramadhani Chambuso, George Rebello, Paul A. Goldberg, Raj Ramesar

**Affiliations:** ^1^ UCT/MRC Genomic and Precision Medicine Research Unit, Division of Human Genetics, Department of Pathology, Institute of Infectious Disease and Molecular Medicine, University of Cape Town and Affiliated Hospitals, Cape Town, South Africa; ^2^ The Colorectal Unit of the Department of Surgery, Groote Schuur Hospital and the University of Cape Town, Cape Town, South Africa

**Keywords:** hereditary colorectal cancer (CRC), next generation sequencing -(NGS), early-onset colorectal cancer, germline variants, genetic insight, indigenous african cohort, South African populations, multigene panel

## Abstract

**Introduction:**

The increase in incidence of colorectal cancer in young patients of African ancestry coupled with increased aggressiveness has warranted investigation of the heritable nature of these cancers. Only a limited number of published reports of hereditary colorectal cancer in indigenous African populations have been reported and no systematic screening of these groups has been performed previously. We aimed to investigate causative germline variants and to establish the incidence of pathogenic/likely pathogenic germline variants in the known colorectal cancer genes in indigenous African colorectal cancer patients using a next-generation sequencing (NGS) multigene panel.

**Materials and methods:**

Patients were selected from two hospitals in Cape Town and Johannesburg, South Africa. Patients with unresolved molecular diagnosis with an age of onset below or at 60 years were selected. Germline DNA samples were analyzed using a 14-gene NGS panel on the Ion Torrent platform. Variant calling and annotation were performed, and variants were classified according to the American College of Medical Genetics and Genomics guidelines. Observed variants were verified by Sanger sequencing and/or long-range PCR.

**Results:**

Out of 107 patients, 25 (23.4%) presented with a pathogenic/likely pathogenic germline variant (PGV). Fourteen PGVs in at least one mismatch repair (MMR) gene were identified and verified in 12 patients (11.2%). Of these MMR gene variants, five were novel. The remaining 10 PGVs were in the APC, BMPR1A, MUTYH, POLD1, and TP53 genes.

**Conclusion:**

The high incidence of PGVs associated with early-onset colorectal cancer in indigenous African patients has important implications for hereditary colorectal cancer risk management. These findings pave the way for personalized genetic screening programs and cascade testing in South Africa. The next step would involve further screening of the unresolved cases using tools to detect copy number variation, methylation, and whole exome sequencing.

## 1 Introduction

Colorectal cancer (CRC) is the third most common neoplastic disease worldwide (1). Approximately 20%–30% of CRC cases are due to inherited pathogenic mutations (2–4). Approximately 10% of all new CRC cases occur in young individuals, and CRC-related mortality has also increased in this group over the past decade (4, 5). It is estimated that approximately 25% of rectal cancers and 10% to 12% of colon cancers will be diagnosed worldwide in people younger than 50 years of age in the next 10 years (4). Notably, in sub-Saharan Africa, the prevalence of CRC is increasing predominantly in young individuals below the age of 40 years especially in indigenous African patients (6–9). Most importantly, comprehensive cancer genetics in early age of onset (eAOO) CRC has not yet been investigated in the indigenous African population despite their increasing prevalence and greater aggressiveness than in non-African populations (10). This presents a public-health problem in cancer genetics. Screening, treatment, and targeted prevention of CRC will remain limited and ineffective in indigenous Africans if this problem is not addressed.

Currently, it is difficult to establish a true picture of hereditary CRC burden in South Africa due to underreporting and underdiagnosis especially among indigenous Africans (11, 12). Consequently, systematic screening and genetic testing strategies for hereditary CRC in these populations have been suboptimal. Only a limited number of published reports of multigene panel testing for hereditary CRC in indigenous Africans have been reported, which remains a major gap in population-specific genetics (13, 14). As we have previously described, South Africa is located at the southern tip of Africa and in a geographic cul-de-sac for southward migration of indigenous Africans along the West and East coasts of the continent (15, 16). In this regard, and anthropologically, our setting in southern Africa provides us with an ideal platform to study the hereditary phenomenon of CRC in indigenous Africans (17, 18). In our ongoing CRC work in local populations in the Western and Northern Cape Provinces of South Africa, we have shown that genetic screening increased the rate of identification of at-risk individuals with inherited cancer-predisposing syndromes including Lynch syndrome (LS) with pathogenic variants in the DNA mismatch repair (MMR) genes (19–21).

For CRC, multigene panel testing by next-generation sequencing (NGS) is now widely used to screen for hereditary CRC. The genetic syndromes associated with CRC are categorized into two distinct groups: non-polyposis and polyposis syndromes, where variants in the non-polyposis syndrome genes are found more prevalent (22). NGS panel testing strategies prioritize inclusion of genes with high prevalence associated with non-polyposis syndromes (23). The selection of genes to include in the NGS panel is based on (i) those most listed as high-risk genes according to the latest American College of Medical Genetics and Genomics (ACMG) guidelines for hereditary CRC, and (ii) genes known as key role players in hereditary CRC previously identified in other populations (24, 25). In addition to the four MMR genes (*MLH1, MSH2, MSH6*, and *PMS2*), the gene *EPCAM* is included in an NGS multigene panel because it has a well-established association with the most common hereditary CRC syndrome, LS, due to deletions in this gene leading to *MSH2* hypermethylation (26–28). The other genes included in the panel are associated with other types of hereditary CRC. These include *APC* for familial adenomatous polyposis (FAP); *BMPR1A* and *SMAD4* for Juvenile Polyposis Syndrome (JPS); *MUTYH for MUTYH*-associated polyposis; *PTEN* for PTEN tumor/hamartoma syndrome (or Cowden syndrome/Bannayan–Riley–Ruvalcaba syndrome); and *STK11* for Peutz–Jeghers syndrome. In addition, *TP53*, which is associated with familial Li–Fraumeni syndrome, and often seen in sporadic cancers, is included in the panel. Recently, studies have shown that *POLD1* and *POLE* play a role in some familial CRC and even in LS predisposition and are also included in our NGS 14-gene panel (29–31). Other genes such as *RPS20* (associated with MMR-proficient, non-polyposis CRC; RPS20-associated hereditary CRC) and *NTHL1* (associated with polyposis hereditary CRC; NTHL1-tumor syndrome) are less prevalent and may be added to CRC NGS panels. There are few other genes reported in the literature such as *RNF43* (associated with serrated polyposis CRC; RNF43-associated serrated polyposis syndrome) and *GREM1* (associated with mixed polyposis CRC; GREM1-associated mixed polyposis) in few affected individuals, but their prevalence is not yet well known and are less common in CRC multigene panel designs (22, 23).

We hypothesize that investigating the germline mutations in affected indigenous African patients with early-onset CRC and establishing the incidence of inherited mutations in the known CRC genes using the NGS 14-gene panel will provide more insights and inferential genetic information on the hereditary nature, treatment options, and targeted prevention of CRC. To investigate our hypothesis in indigenous African patients, we aimed to determine causative germline variants in CRC patients of indigenous African descent, and to establish the incidence of pathogenic or likely pathogenic germline variants in the known CRC genes in our indigenous African cohort, using an NGS multigene panel, in South Africa.

## 2 Materials and methods

### 2.1 Research ethics

All procedures were performed in accordance with guidelines of the Declaration of Helsinki. Ethical approval for the study was granted by the Human Research Ethics Committee of (i) the University of Cape Town (UCT), ethics approval number HREC:287/2020; (ii) all respective hospitals ethics departments; and (iii) the South African National Cancer Registry (NCR). Consent forms were previously provided to the study participants to be involved in genetic research before data collection and storage. The patients were counseled by a registered practitioner who explained the details of the research and answered all questions from the patients, in their home language, as part of the informed consent procedure.

### 2.2 Selection of patient cohort

Our study participant selection criteria were as follows: (i) diagnosed CRC patients of indigenous African origin, (ii) age of onset (or diagnosis age) below or equal to 60 years, (iii) available genomic DNA samples, and (iv) no known molecular diagnosis for CRC for a genetic/germline variant. Selection of these patients of indigenous African origin for our study cohort was based on self-identification criteria that patients note in their records in our hospital recruitment in South Africa, as also required by the NCR in the country^1^ (where the cancer is a notifiable disorder). As part of a large ongoing hospital-based project on the genetics of CRC, a total of 107 DNA samples collected over the past 30 years were included in this study. In our cohort, we selected DNA samples from Groote Schuur Hospital in Cape Town whose records were stored in the Division of Human Genetics repository (*n* = 64), which contains clinical information and matching molecular test results. We also selected DNA samples from the Chris Hani Baragwanath Hospital in Johannesburg whose records were stored at the South African NCR database (*n* = 43), which also contains clinical information and molecular test results. All CRC patients were previously admitted at these two hospitals in the last three decades.

### 2.3 Sample preparation

#### 2.3.1 Initial quality control

DNA samples from peripheral blood were previously extracted using basic salting-out methodology according to adopted protocol in the Division of Human Genetics Laboratory at UCT and were subjected to an initial quality control (QC) to evaluate DNA dilutions before NGS screening (32). The QC involved stock DNA quantification through spectrophotometry using a Nanodrop ND 1000 spectrophotometer (Applied Biosystems, Thermo Fisher Scientific, Massachusetts, USA) and an integrity check using agarose gel electrophoresis. Samples with a concentration lower than 50 ng/µL, those that did not meet the DNA purity (e.g., A_260/280_ ratio), and degraded samples were excluded.

#### 2.3.2 Extended quality control for NGS library preparation with Qubit HS and real-time PCR

A second QC step was implemented for specifically the NGS library preparation. The selected samples were diluted to 1 ng/µL for NGS library preparation. These dilutions were quantified using Qubit dsDNA High Sensitivity assay (Applied Biosystems, Thermo Fisher Scientific, Massachusetts, USA) followed by a real-time PCR using a TaqMan™ RNase P Detection Reagents Kit (Applied Biosystems, Thermo Fisher Scientific, Massachusetts, USA) according to the manufacturer’s instructions. Those with insufficient concentration and quality were excluded from NGS sequencing.

### 2.4 Next-generation sequencing panel screening

Library preparation of the NGS 14-gene panel for hereditary CRC (*APC*, *BMPR1A, EPCAM, MLH1, MSH2, MSH6, MUTYH, PMS2, POLD1, POLE, PTEN, SMAD4, STK11*, and *TP53*) was performed using the Ion AmpliSeq Kit for Chef DL8 according to the manufacturer’s instructions (Applied Biosystems, Thermo Fisher Scientific, Massachusetts, USA). The panel was previously designed in our laboratory based on the existing literature and experiences in our CRC research. The list of the genes and their molecular genetic characteristics is provided in **Table 1**. Fifteen microliters of each diluted DNA sample (total DNA yield of 15 ng) of DNA was loaded into each well on the library DL8 plate on the Ion Chef instrument for automated Ion AmpliSeq library construction instrument (Thermo Fisher Scientific, Massachusetts, USA). The cycling conditions were set up as the following: the number of primer pairs per pool, 193–384; number of amplification cycles, 18; and anneal/extension time of 4 min. After library preparation, library quantitation was performed using an Ion Library TaqMan Quantitation Kit according to the manufacturer’s instructions (Thermo Fisher Scientific, Massachusetts, USA). The cycling conditions for library quantitation involved Uracil DNA glycosylase incubation at 50°C for 2 min, a polymerase activation at 95°C for 3 s and 40 cycles at 60°C for 30 s. The libraries were then diluted according to the quantification results after the qPCR, to a concentration ±100 pM. Libraries were then pooled, and the pooled libraries were quantified by using the same qPCR protocol as for library quantitation used for each library. The pooled libraries were loaded onto the Ion 520™ Chips (Applied Biosystems, Thermo Fisher Scientific, Massachusetts, USA) for templating on the Ion Chef instrument. Finally, sequencing was performed on an S5 sequencer (Applied Biosystems, Thermo Fisher Scientific, Massachusetts, USA).

**Table 1:** List of the 14 genes included in the next-generation sequencing panel and their reference sequence along with the chromosomal locations, risk levels, and the phenotypes.

Gene	RefSeq	Chr locus	Risk level^a^	Phenotype/Disease (ClinGen)
*APC: Adenomatous polyposis coli*	NM_000038.6	5q22.2	High	Familial adenomatous polyposis (FAP)/attenuated FAP Gastric adenocarcinoma and proximal polyposis of the stomach
*BMPR1A: Bone Morphogenetic Protein Receptor Type 1A*	NM_004329.3	10q23.2	High	Juvenile polyposis syndrome Hereditary mixed polyposis syndrome Pulmonary arterial hypertension
*EPCAM: Epithelial cellular adhesion molecule*	NM_002354.3	2p21	High	Lynch syndrome
*MLH1: MutL, E. coli, homolog of, 1*	NM_000249.4	3p21.3	High	Lynch syndrome, mismatch repair cancer syndrome 1 Hereditary breast carcinoma
*MSH2: MutS, E. coli, homolog of, 2*	NM_000251.3	2p22-p21	High	Lynch syndrome, mismatch repair cancer syndrome 1 Hereditary breast carcinoma
*MSH6: MutS, E. coli, homolog of, 6*	NM_000179.3	2p16	High	Lynch syndrome, mismatch repair cancer syndrome 1 Hereditary breast carcinoma
*MUTYH: MutY DNA glycosylase*	NM_001128425.2	1p34.1	High	MUTYH-associated polyposis FAP II
*PMS2: Postmeiotic segregation increased S. cerevisiae*	NM_000535.7	7p22	High	Lynch syndrome Mismatch repair cancer syndrome I Hereditary breast carcinoma
*POLD1: Polymerase (DNA directed), delta1, catalytic subunit*	NM_001256849.1	19q13.33	Risk	Polymerase proofreading-associated polyposis
*POLE: Polymerase, DNA, epsilon*	NM_006231.4	12q24.33	Risk	Polymerase proofreading-associated polyposis
*PTEN: Phosphatase and tensin homolog*	NM_000314.8	10q23.31	High	PTEN hamartoma tumor syndrome (or Cowden syndrome/Bannayan–Riley–Ruvalcaba syndrome)
*SMAD4: SMAD family member 4*	NM_005359.6	18q21.2	High	Juvenile polyposis syndrome (JPS); JPS/hereditary hemorrhagic telangiectasia syndrome
*STK11: Serine/threonine kinase 11*	NM_000455.5	19p13.3	High	Peutz–Jeghers syndrome
*TP53: Tumor protein p53*	NM_000546.5	17p13.1	Lower^b^	Li–Fraumeni syndrome

^a^ACMG-inherited CRC guidelines, 2021 (24). “^b^Recommendation: “Genes associated with hereditary cancer syndrome where CRC/polyposis are possible manifestations associated with defined hereditary cancer predisposition syndromes, which include CRC and/or polyposis as rare manifestations. As it is difficult to separate out specific CRC/polyposis risk, it is reasonable toinclude these genes routinely on multigene panels for hereditary CRC.”

### 2.5 Variant annotation and filtering

The sequencing data were processed through the Ion Server software and read alignment was done against the *Homo sapiens* reference genome hg19 to create BAM files. Variant calling and annotation were performed on the Ion Reporter software^2^. The *tsv* output files generated for each patient were merged into a single Microsoft Excel file for variant filtering. The report included depth of coverage for each variant, minor allele frequency (MAF), location, function, four different pathogenicity prediction estimates (Sift, Polyphen, Grantham, and FATHMM), and information in some public archives of reports on variants and their phenotypes such as ClinVar^3^ and dbSNP^4^.

**Figure 1:**
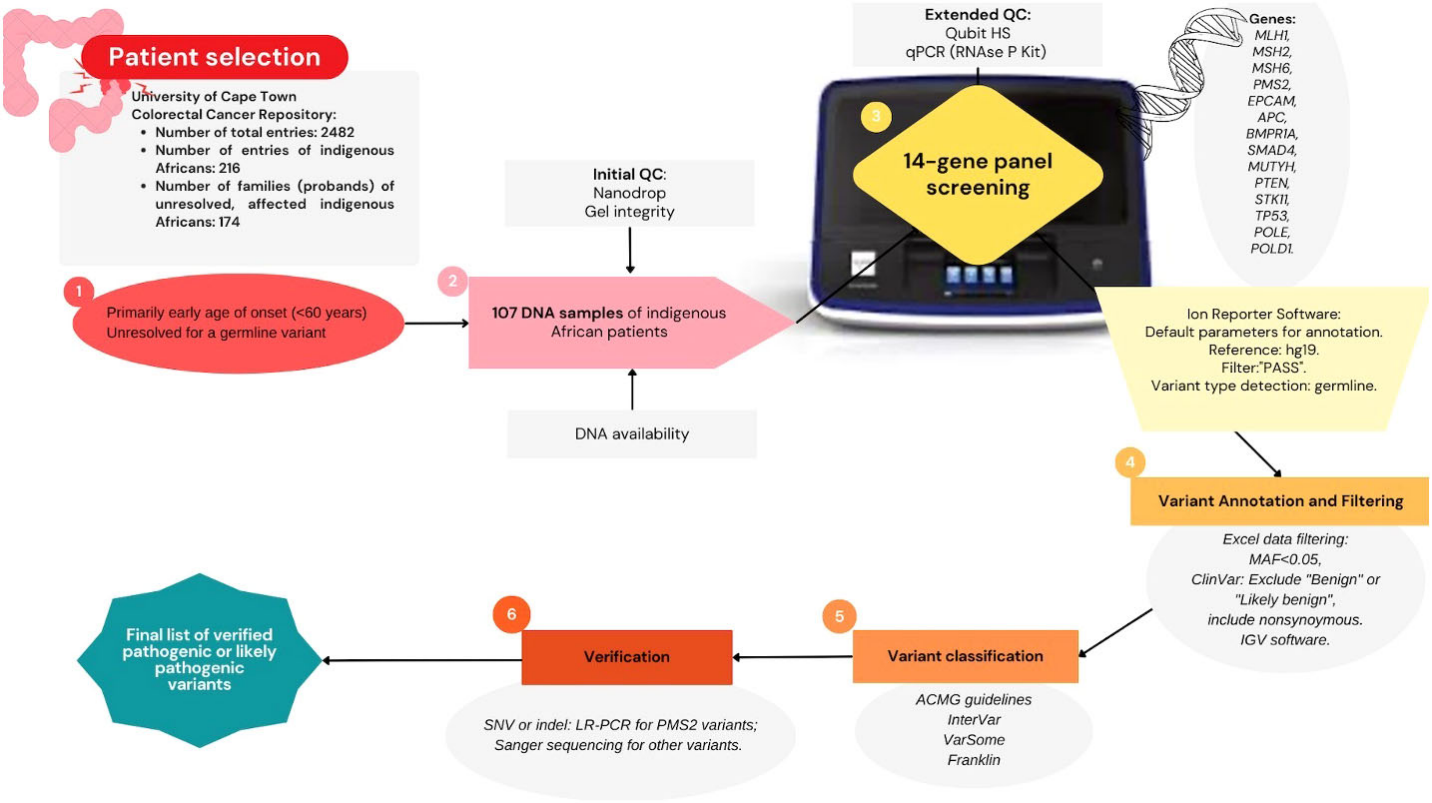
Summary diagram of methodology: Next-generation sequencing multigene panel screening workflow; starting from applying selection criteria to prioritize a colorectal cancer patient cohort (*n* = 107), quality control assessment, sequencing performed on the Ion Torrent platform to generate germline variant data, followed by data filtering, variant classification, and verification.

The Ion Reporter software only generated output files of variants labeled as “PASS”, and these were subjected to filtering. All synonymous variants were excluded and only exonic variants and variants in the flanking regions with an MAF less than 0.05 were retained. The MAF <0.05 was considered as the cutoff for potential rare variants to ensure any population specific variant may not be missed. These variants were then prioritized for further analysis. The data were not filtered based on depth of coverage, but each prioritized variant was manually inspected for the depth of coverage. Variants with 30× coverage or less were excluded from the prioritized list. Variants that were classified as pathogenic, likely pathogenic, uncertain significance, conflicting interpretation, or with a blank record on ClinVar were selected and further filtered by manual inspection. The prioritized variants, after the filtering, were then manually inspected on the Integrative Genomics Viewer (IGV) software to assess depth of coverage and integrity of base calls (33). Some calls were found to be an artifact of random insertions or deletions around the region where the variant is located despite a high coverage, present in multiple samples, and these were excluded from the prioritized list. VarSome, Franklin,^5^ and InterVar were used to help in assigning variant classification, according to the ACMG guidelines (34, 35). Variants classified as pathogenic or likely pathogenic were selected for verification. The workflow for the NGS panel screening from patient selection to verification is summarized in **Figure 1**.

### 2.6 Verification of the pathogenic or likely pathogenic germline variants

#### 2.6.1 Variant verification by Sanger sequencing

Primers were either used from available stock or newly designed for the variants that needed verification by Sanger sequencing. Basic PCR amplification for each variant was then performed using GoTaq^®^ Flexi DNA Polymerase kit according to the manufacturer’s instructions (Promega Corporation, Wisconsin, USA). To purify the PCR products prior to the sequencing reaction, they were subjected to an enzymatic clean-up reaction by the FastAP (Thermosensitive alkaline phosphatase) and *ExoI* (Exonuclease I) enzymes according to the manufacturer’s instructions (Promega Corporation, Wisconsin, USA). PCR products were incubated at 37°C for 1 h followed by 75°C for 15 min.

The presence of the prioritized pathogenic or likely pathogenic germline variants was then verified by Sanger sequencing using BigDye™ Terminator v3.1 (BDT V3.1) Cycle Sequencing Kit (Applied Biosystems, Thermo Fisher Scientific, Massachusetts, USA) according to the manufacturer’s instructions. Sequencing reactions were purified using ethanol precipitation prior to capillary electrophoresis, which was carried out on the ABI 3130xl and the SeqStudio genetic analyzers (Applied Biosystems, Thermo Fisher Scientific, Massachusetts, USA). Base calling was performed on the Sequencing Analysis v5.4 software (Applied Biosystems, Thermo Fisher Scientific, Massachusetts, USA). Sequences were visualized on MySequence application.^6^

#### 2.6.2 Variant verification by long-range PCR

A protocol for long-range PCR (LR-PCR) with a “long and accurate” *Taq* polymerase (TaKaRa Bio Inc., Otsu, Shiga, Japan) was optimized, similar to other studies, and used to confirm the presence of the variants found in the regions of the *PMS2* gene that are in complete homology with the known pseudogene that interferes with the coding *PMS2* variant interpretation (36–38). The observed *PMS2* variants were in the 3’ end of *PMS2*, which is also contained in the pseudogene, *PMS2CL*. DNA samples were diluted to 50 ng/µL, and 2 µL (making a total DNA yield of 100 ng) was used in a total volume of a 25-µL LR-PCR reaction. Previously published primers were used in a final concentration of 0.2 µM (39, 40). The cycling involved an initial denaturation at 94°C for 1 min, followed by 35 cycles of 94°C for 15 s, 65°C for 30 s, and 68°C for 15 min. Final elongation was set at 72°C for 10 min. The LR products were electrophoresed on a 0.4% agarose gel for confirmation before proceeding with the second PCR. Lambda DNA/*Hin*dIII Marker (Applied Biosystems, Thermo Fisher Scientific, Massachusetts, USA) was used as a high-molecular-weight marker when visualizing the LR-PCR products. Each LR-PCR product was diluted with 1:10 ratio and the diluted LR-PCR products were used as a template for the second, exon-specific PCR. This was carried out using GoTaq^®^ Flexi DNA Polymerase according to the manufacturer’s instructions (Promega Corporation, USA) in a final reaction volume of 25 μL. Primers for the second PCR amplifications were designed according to the long-range PCR product’s expected reference sequence. Finally, Sanger sequencing was performed using the second PCR products following the same protocol as in the Sanger sequencing verification above.

#### 2.6.3 Variants of uncertain significance analysis in the NGS data

The total number of variants of uncertain significance (VUSs) was calculated from the unfiltered NGS panel data files of the 107 samples. All the *tsv* output files were compiled into a single *tsv* file using R Studio, which was then filtered in Excel. The VUSs were filtered on the “ClinVar” column in the Excel sheet (41). Further filtering criteria were applied following the same parameters as the filtering criteria for pathogenic/likely pathogenic germline variant (PGV) filtering; synonymous and non-exonic variants with a depth of coverage less than 30× and with MAF greater than 0.05 were excluded.

### 2.7 Statistical analysis

The number of patients with PGVs was divided by the total number of patients analyzed to get the descriptive statistics. Pearson’s Chi-squared test or Fisher’s exact test were used to compare categorical variables. PGVs were used as dependent variables for testing the variant pathogenicity variations between different predictor variables such as age of onset, gender, and tumor location or sidedness of each patient. A *p*-value less than 0.05 was considered significant. The analyses were conducted using R Studio (version 2021.9.1.372) (41).

## 3 Results

### 3.1 Patient cohort and characteristics

Overall patients’ demographics and clinical–pathological characteristics are summarized in **Table 2**. Patients’ clinical features were statistically compared to their mutation status (positive/negative) which did not show any statistical significance. The median age of the mutation-positive and the mutation-negative patients was similar: 35 and 37 years old, respectively (*p*-value = 0.630). The youngest and the oldest age at diagnosis were 26 and 58 years, respectively (median = 37). The number of male and female patients were nearly equal: 54 and 53 years, respectively. Tumor was predominantly localized in the colon with nearly 74% of the total cohort, and the ratio of the right-sided and left-sided tumors was 50% in the overall cohort. Only one and six patients lacked clear information about the tumor localization and sidedness, respectively. Available information on previous immunohistochemistry (IHC) testing result for patients in our database was also retrieved. Although these samples were only a handful of the total indigenous African cohort, it helped us make the comparison between the MMR PGVs versus patients’ IHC status (23 out of 107) (**Supplementary Table 1**).

**Table 2:** Patient demographics and clinical–pathological characteristics.^a^

	All (*N* = 107)	Positive (*N* = 27)	Negative (*N* = 80)	*p*-value
**Total:**	107	25.23%	74.77%	
Diagnosis age (years)				
Mean (SD)^b^	35.64 (8.71)	35.33 (9.24)	35.76 (8.58)	
Median	37	35	37	0.630^c^
Range	26 to 58	26 to 58	18 to 58	
Categorical age				
<50 years	104 (97.20%)	25 (92.59%)	79 (98.75%)	0.156^d^
≥50 years	3 (2.80%)	2 (7.41%)	1 (1.25%)	
Gender				
Male	54 (50.94%)	15 (55.56%)	39 (48.75%)	0.697^e^
Female	53 (49.06%)	12 (46.15%)	41 (50.62%)	
Tumor localization				
Colon	79 (73.83%)	18 (66.67%)	61 (76.25%)	0.069^d^
Rectum	25 (23.36%)	7 (25.93%)	18 (22.50%)	
Both	2 (1.87%)	2 (7.41%)	0	
Not applicable	1 (0.93%)	0	1 (1.25%)	
Tumor sidedness				
Right sided	47 (43.93%)	12 (44.44%)	35 (43.75%)	0.144^d^
Left sided	47 (43.93%)	10 (37.04%)	37 (46.25%)	
Both	7 (6.54%)	4 (14.81%)	3 (3.75%)	
Not applicable	6 (5.61%)	1 (3.70%)	5 (6.25%)	
Immunohistochemistry testing status				
Loss of a mismatch repair protein	14 (13.08%)	5 (18.52%)	9 (11.25%)	
Normal staining (no loss)	9 (8.41%)	3 (11.11%)	6 (7.50%)	
Not applicable	84 (78.50%)	19 (70.37%)	66 (82.50%)	

^a^This table includes all the patients presented with a pathogenic/likely pathogenic variant before the verification step. ^b^Standard deviation. ^c^Wilcoxon rank-sum test. ^d^Fisher’s exact test. ^e^Pearson’s Chi-squared test.

### 3.2 Variant filtering results

Variant detection was performed using the NGS multigene panel for the DNA samples that passed the QC. Mean depth of coverage of the complete targeted regions in the NGS data was 604×. The total number of variants detected, before any filtering, was 15,063 (100 to 200 per individual). After data filtering based on the location and the function of variants, this number decreased to 1,092 (10–15 variants per individual). Further filtering out of variants with a MAF (>0.05) then reduced the total number of retained variants to 360, and duplicates were removed (305 unique variants), which ranged from zero to eight variants per individual, for further inspection for variant classification. The final prioritized list of patients presented with a PGV, before verification, and their features are shown in **Table 3** (see **Supplementary Table 2** for detailed information).

**Table 3:** List of patients with germline pathogenic and/or likely pathogenic variants that were identified after the NGS multigene panel screening in this study.

Sample ID	Gene	Coding DNA	Protein	MAF^a^	ClinVar	ACMG^b^ variant classification	Reported before?
***1*** *^c^*	*MLH1*	c.117-1G>A	p.?	NA	Pathogenic	Pathogenic	No (G>T only)
***2*** *^d^*	*MLH1*	c.2263A>G	p.Arg755Gly	NA	Pathogenic	Pathogenic	No
***3***	*MLH1*	c.117-1G>A	p.?	NA	Pathogenic	Pathogenic	No (G>T only)
***4***	*MLH1*	c.793C>T	p.Arg265Cys	NA	Pathogenic	Pathogenic	Yes
***5*** *^d^*	*BMPR1A*	c.834C>A	p.Tyr278Ter	NA	Pathogenic	Pathogenic	No
***6*** *^d^*	*BMPR1A*	c.717delA	p.Val240LeufsTer21	NA	No entry	Pathogenic	No
***7***	*MSH2*	c.2377C>T	p.Gln793Ter	NA	Pathogenic/likely pathogenic	Pathogenic	No
***8*** *^d^*	*POLD1*	c.1816C>A	p.Leu606Met	NA	Conflicting interpretation of pathogenicity	Likely pathogenic	Yes (*de novo* in one African)
***9***	*BMPR1A*	c.185A>G	p.Tyr62Cys	NA	Uncertain significance	Likely pathogenic	No
***10***	*PMS2*	c.2192_2196delTAACT	p.Leu731CysfsTer3	0.0	Pathogenic	Pathogenic	Yes
***11***	*BMPR1A*	c.717delA	p.Val240LeufsTer21	NA	No entry	Pathogenic	No
***12*** *^c^*	*MSH2*	c.187delG	p.Val63Ter	NA	Pathogenic	Pathogenic	Yes (breast cancer)
***13***	*TP53*	c.526T>C	p.Cys176Arg	NA	Conflicting interpretation of pathogenicity	Pathogenic	Yes (only somatic)
**14**	*POLD1*	c.1265_1266insG	p.Arg423ProfsTer212	NA	No entry	Pathogenic	No
***15***	*BMPR1A*	c.355C>T	p.Arg119Cys	NA	Conflicting interpretation of pathogenicity	Pathogenic	Yes
***16***	*MSH2*	c.1705_1706delGA	p.Glu569IlefsTer2	NA	Pathogenic	Pathogenic	Yes
***17*** *^c^*	*MSH2*	c.387_388delTC	p.Gln130fs	NA	Pathogenic	Pathogenic	Yes
***18***	*PMS2*	c.1579_1580delAG	p.Arg527GlyfsTer14	0.0	Pathogenic	Pathogenic	Yes (breast/ovarian cancer)
***19***	*MSH2*	c.643C>A	p.Gln215Lys	NA	No entry	Likely pathogenic	No (C>T only)
***20^d^***	*MLH1*	c.1731G>A	p.Ser577=	NA	Pathogenic	Pathogenic	Yes
***21***	*MSH2*	c.1923T>A	p.Cys641Ter	NA	No entry	Pathogenic	No
***21***	*PMS2*	c.2192_2196delTAACT	p.Leu731CysfsTer3	0.0	Pathogenic	Pathogenic	Yes
***22***	*PMS2*	c.2192_2196delTAACT	p.Leu731CysfsTer3	0.0	Pathogenic	Pathogenic	Yes
***23***	*MSH2*	c.187_188insG	p.Val63fs	NA	Pathogenic	Pathogenic	Yes
***24***	*MUTYH*	c.1476 + 2C>T	p.?	0.002	Conflicting interpretations of pathogenicity	Likely pathogenic	Yes
***25***	*MSH2*	c.1569delT	p.Arg524ValfsTer2	NA	Pathogenic	Pathogenic	Yes
***26***	*TP53*	c.742C>T	p.Arg248Trp	NA	Pathogenic	Pathogenic	Yes
**27**	*APC*	c.2868C>A	p.Tyr956Ter	NA	No entry	Likely pathogenic	No (C>G only)

^a^Minor allele frequency, gnomAD. “NA” = “Not available”. ^b^American College of Medical Genetics and Genomics (42). ^c^Family history of cancer. ^d^Deceased after diagnosis.

### 3.3 Pathogenic/likely pathogenic germline variants identified after the NGS panel screening

**Figure 2:**
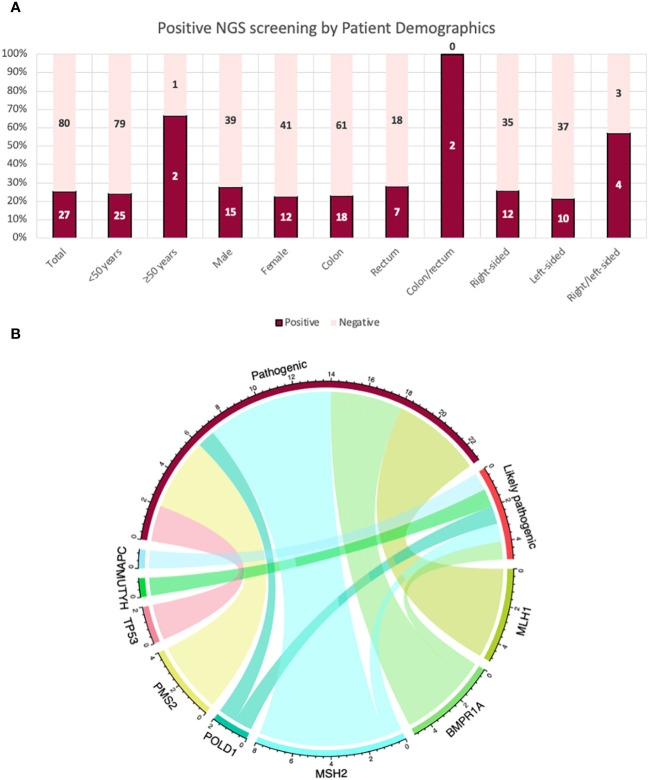
**(A)** Patients with a pathogenic/likely pathogenic variant post-NGS 14-gene panel analysis by their characteristics and demographics. **(B)** A chord diagram to demonstrate the relationship of the genes for pathogenicity (pathogenic and likely pathogenic variants) in the total cohort in this study: Each bar represents a variant in their corresponding genes; aligning with its corresponding type of variant as either pathogenic or likely pathogenic. The highest proportion of variants were in *MSH2* (*n* = 8); seven pathogenic variants and one likely pathogenic variant.

**Figure 3:**
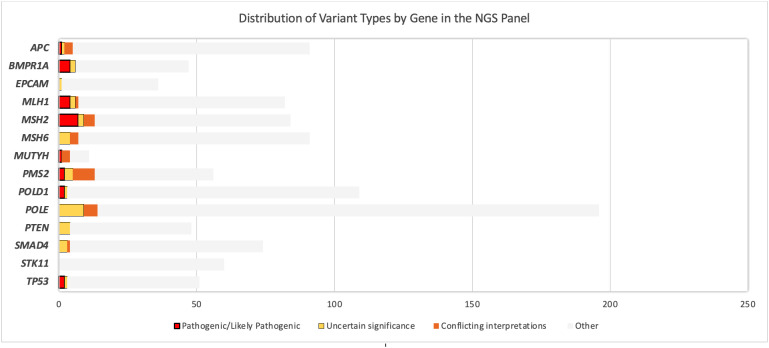
Distribution of the variants per gene in the next-generation sequencing 14-gene panel used in this study: with variant types of pathogenicity. *EPCAM*, *PTEN*, *SMAD4*, and *STK11* did not carry any pathogenic/likely pathogenic variant (PGV) or any variant of uncertain significance (VUS). “Other” includes variants besides PGV and VUS (e.g., benign/likely benign variants, variants not reported in ClinVar).

The NGS multigene panel screening resulted in 24 germline PGVs in 27 patients, before verification. Fourteen PGVs in the MMR genes were identified: four pathogenic variants in *MLH1*, seven pathogenic variants and one likely pathogenic variant in *MSH2*, two pathogenic variants in *PMS2*, and no PGV in *MSH6*. The remainder of the observed germline variants were in the *APC* (one likely pathogenic variant), *BMPR1A* (two pathogenic and two likely pathogenic variants), *MUTYH* (one likely pathogenic variant), *POLD1* (one pathogenic and one likely pathogenic variants), and *TP53* (two pathogenic variants) genes, shown in **Figure 2**. Ten of the observed PGVs were novel; 50% of these were MMR variants. The distribution of the variants per gene in the NGS 14-gene panel used in this study with variant types of pathogenic, likely pathogenic, and VUS is summarized in **Figure 3** where *STK11* did not carry any of the three types of variants, and *EPCAM* and *PTEN* only contained VUSs.

#### 3.3.1 Variants identified in MMR genes

An *MLH1 (NM_000249.4)* missense pathogenic variant, c.2263A>G, was found in one individual, patient 2, whose age at diagnosis was less than 30 years. Another pathogenic *MLH1* variant, located at canonical splice site (*MLH1*:c.117-1G>A), was identified in two individuals, patient 1 and patient 3, who were both diagnosed under 40 years of age. Another pathogenic variant in *MLH1*, c.793C>T, was carried by another individual, patient 4, diagnosed at under 50 years of age; this variant has been previously reported in literature in affected CRC patients (43–48). Importantly, manual inspection allowed us to identify two additional pathogenic MMR variants: one was a synonymous, but pathogenic splice site variant in *MLH1*, c.1731G>A in patient 20. This patient lacked detailed clinical information but was diagnosed under 50 years of age and the records indicated that the patient deceased only a few months after diagnosis. The other pathogenic variant was a two-base pair deletion, *MSH2*:c.387_388delTC in patient 17 (eAOO <30 years) who had a family history of cancer (**Table 3**; **Supplementary Figure 1**).

An insertion in the first exon of *MSH2 (NM_000251.3)*, c.187_188insG, was present in patient 23, who was diagnosed at under 40 years of age. A deletion was present at the same position; *MSH2*:c.187delG in another individual (patient 12); these both cause an early protein truncation. Both deletion and duplication at position 187 have previously been reported in LS, breast, endometrial, or ovarian cancer patients and affected family members (49–52). A frameshift pathogenic variant in *MSH2*; c.1705_1706delGA was present in one individual and has also been reported in the literature before as a disease-causing variant associated with CRC (53). A novel pathogenic variant, *MSH2*:c.1569delT, was found in one patient, patient 25, with strong evidence for pathogenicity according to the ACMG guidelines (42).

One patient presented with two distinct pathogenic germline variants in the MMR genes *MSH2* and *PMS2 (NM_000535.7)*, after variant filtering and manual inspection. The *MSH2*:c.1923T>A variant identified in this individual (patient 21) is a novel, stop-gain variant in exon 12 (which has a total of 16 exons). The *PMS2*:c.2192_2196delTAACT variant is also a pathogenic, previously reported, frameshift variant. However, LR-PCR did not verify the presence of the *PMS2* variant in the patient.

#### 3.3.2 Variants identified in the non-MMR genes in the NGS panel

Two pathogenic variants in *TP53 (NM_000546.6)*, *TP53*:c.526T>C and *TP53*:c.742C>T, were present in two unrelated patients, patient 13 and patient 26, respectively. These patients were both diagnosed at a relatively early age (less than 30 years). A pathogenic variant in *APC (NM_000038.6)*, *APC*:c.2868C>A, was identified in another patient, patient 27, and this finding corresponded with the pathology report providing clinical information associated with FAP. A frameshift *POLD1 (NM_001256849.1)* pathogenic variant, c.1265_1266insG, which leads to protein truncation in exon 11 (total exon number = 27), was carried by patient 14 who was diagnosed at a relatively early age (younger than 25 years), also having rectal lesions. Insufficient clinical information was available on this patient to supply further information. A likely pathogenic variant was present in another non-MMR gene, *MUTYH (NM_001128425.2);* c.1476 + 2C>T, in patient 24, where the patient presented with polyps and right-sided tumor that spread to the liver, which is consistent with a polyposis (or *MUTYH*) associated CRC.

Five unrelated patients carried a PGV in *BMPR1A (NM_004329.3)*. One of these patients (patient 5) carried a pathogenic variant, *BMPR1A:*c.834C>A, and this was consistent with the clinical diagnosis of the patient, i.e., the patient was clinically diagnosed with JPS, but no mutation had been identified to date. This patient was less than 40 years of age when diagnosed and died a year after diagnosis. Another pathogenic, frameshift variant, *BMPR1A*:c.717delA, was found in two unrelated individuals (patient 6 and patient 11) who were diagnosed at age younger than 30 and 40 years, respectively. Lastly, two likely pathogenic *BMPR1A* variants were also present in two unrelated individuals, *BMPR1A*:c.185A>G in patient 9 and *BMPR1A*:c.355C>T in patient 15. These patients, patient 9 and patient 15, were also diagnosed at relatively young ages, i.e., <40 and <30 years, respectively. Some of the patients who were recorded to have an MMR protein loss based on their previous IHC testing presented with either an MMR or a non-MMR variant in this study, and age range at diagnosis is also shown, for comparison purposes as in **Table 4**. Among these, only six of the IHC testing results corresponded with the NGS panel screening result. Interestingly, two individuals previously had a normal IHC staining of all the MMR genes, and the NGS panel screening resulted in a PGV in a non-MMR gene, *POLD1*, which supports the IHC finding. Another patient was IHC-negative for *MSH2* and *MSH6* and was found to carry a germline variant only in *MSH2*. MSH2 deficiency was probably the first event followed by the MSH6 deficiency afterwards. This was consistent with the IHC test result for MSH2, but *MSH6* needs to be further explored. Another patient previously presented with loss of MLH1; however, a likely pathogenic variant was present in *MSH2*, but no variant was detected in *MLH1*. Either MSH2 is not the cause or IHC staining for MLH1 might be incorrect, or we did not find the *MLH1* mutation yet (e.g., a copy number variant, an intronic change, or MLH1 methylation). There were nine patients without any germline variant identified after the NGS panel screening although they previously presented with evidence for loss of at least one MMR gene through IHC testing.

**Table 4:** List of patients from our mutation-positive cohort whose previous immunohistochemistry (IHC) testing was available in our database.

Sample ID	IHC staining information	Variant identified	Age (years)
***1***	MLH1 negative	*MLH1:*c.117-1G>A	<40
***8***	All positive	*POLD1:*c.1816C>A	<30
***14***	All positive	*POLD1:*c.1265_1266insG	<30
***16***	MSH2 and MSH6 negative	*MSH2:*c.1705_1706delGA	<60
***17***	MSH2 negative	*MSH2:c.387_388delTC*	<30
***19***	MLH1 negative	*MSH2:*c.643C>A	<30
***20***	MLH1 and PMS2 negative	*MLH1:c*.1731G>A	<50

### 3.4 Verification of the observed germline variants

**Figure 4:**
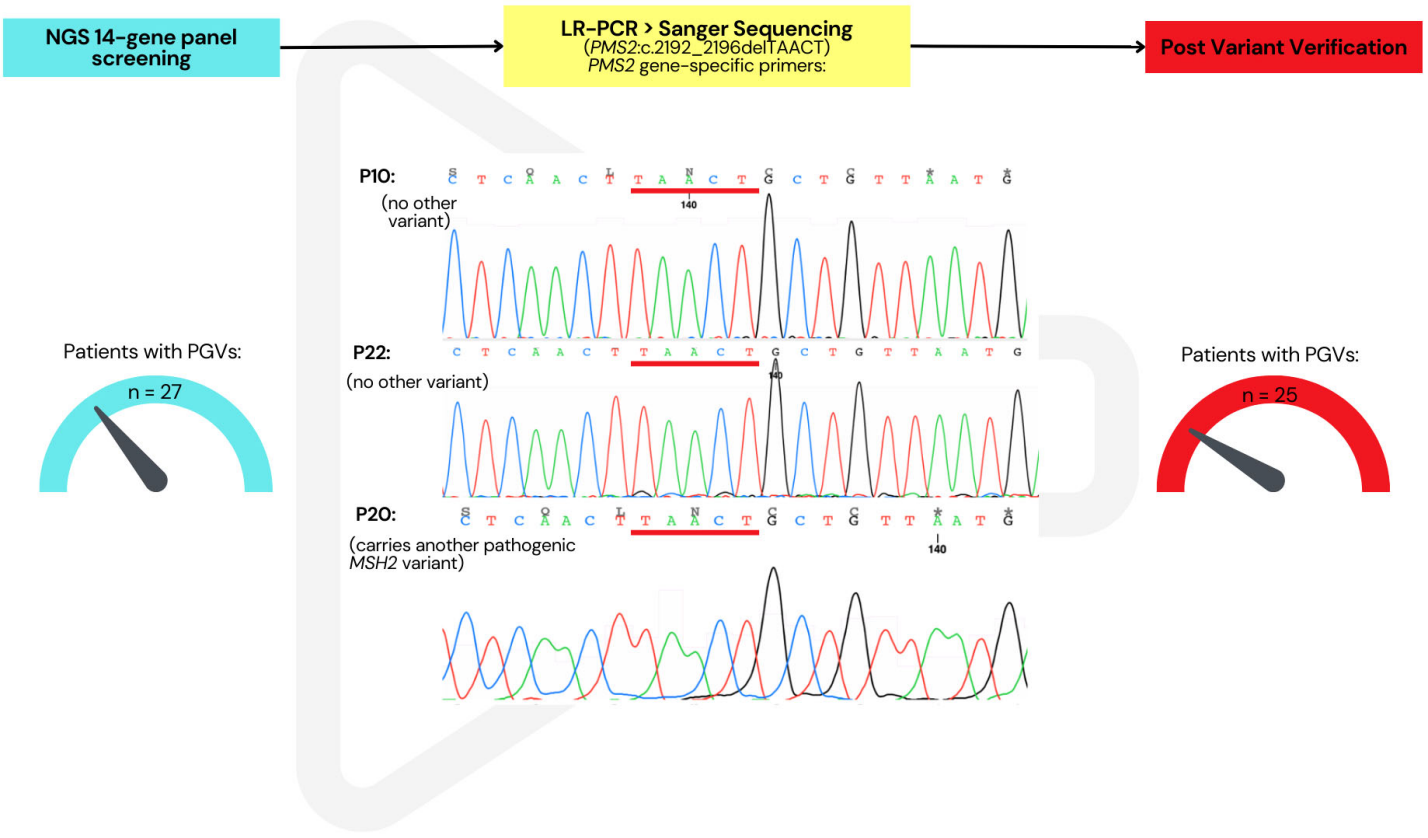
Verification of *PMS2* variants by long-range PCR followed by Sanger sequencing. *PMS2*:c.2192_2196delTAACT not present in the three samples but originally indicated in the NGS panel analysis. After verification, this reduced the total number of patients with a pathogenic/likely pathogenic germline variant from 27 to 25.

Sanger sequencing and/or LR-PCR were successful in verifying the presence of all the observed variants, except for one of the *PMS2* variants. LR-PCR was performed for two *PMS2* variants, followed by a second PCR and Sanger sequencing. The variant *PMS2*:c.1579_1580delAG was present in one sample, successfully verified by LR-PCR (**Supplementary Figure 2**). However, LR-PCR verification of the *PMS2*:c.2192_2196delTAACT variant showed that this variant was not present in *PMS2* in three patients. The status of the patients who only presented with this “unverified” variant (*n* = 2) remained “unresolved” in our database after this study (**Figure 4**). Further methodologies are being investigated to finalize the search for disease-causing genetic changes of these cases. Consequently, the number of verified PGVs was 24, found in 25 patients (23.4%).

### 3.5 VUS identified after the NGS panel screening

**Figure 5:**
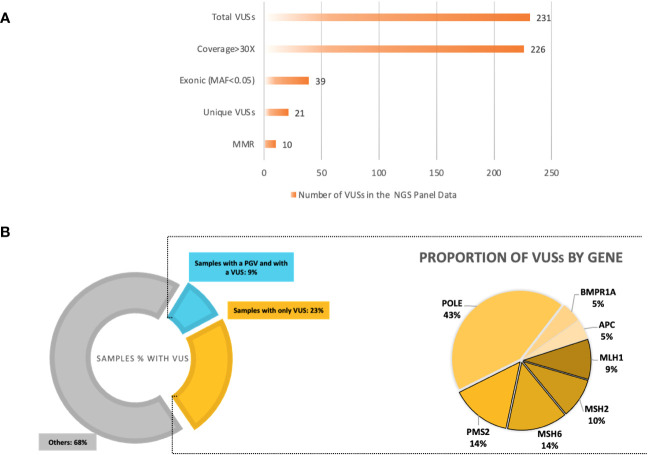
**(A)** Variants of uncertain significance (VUSs) based on ClinVar entry from the next-generation sequencing (NGS) 14-gene panel output data. A total of 232 VUSs were observed; the total number of unique, exonic VUSs with a MAF<0.05 and coverage >30× was 23 and 50% of these were in mismatch repair (MMR) genes, excluding *EPCAM*. **(B)** Left: The proportions of samples with a VUS in the total cohort; >30% of our cohort with a VUS, samples with only a VUS being 23%, and samples with already a pathogenic/likely pathogenic variant who also carries a VUS are 9%. Right: The percentages of VUSs by gene in the NGS 14-gene panel data of the total cohort. The MMR VUSs are highlighted with borders, comprising 50% of the total number of VUSs.

The total number of VUSs called, based on the ClinVar entries on the Ion reporter, was 231. Filtering out the VUSs with a coverage less than 30× dropped this to 226. Out of these, 39 VUSs were in an exonic region; after removing duplicates, the final number of unique VUSs was 21 (MAF < 0.05 or not available), present in 35 patients (32.71%) (**Table 5**; **Supplementary Table 3**). Ten of these unique VUSs were in an *MMR* gene; two in *MLH1*, two in *MSH2*, three in *MSH6*, and three in *PMS2*, carried by 19 patients (19/107 = 17.8%). Nine of the 35 patients who carried a VUS were already found with a PGV (**Figure 5**).

**Table 5:** List of prioritized variants of uncertain significance identified after the NGS multigene panel screening in this study.

Gene	Location	MAF^a^	Function	Coding	Protein
**MMR^b^ genes**	*MLH1*:exonic:NM_000249.3	0.0	Missense	c.1013A>G	p.Asn338Ser
	*MLH1*:exonic:NM_000249.4	NA^c^	Missense	c.1772A>G	p.Asp591Gly
	*MSH2*:exonic:NM_000251.3	NA	Missense	c.508C>G	p.Gln170Glu
	*MSH2*:exonic:NM_000251.3	NA	Missense	c.157G>T	p.Ala53Ser
	*MSH6*:exonic:NM_000179.3	NA	Missense	c.560A>G	p.Lys187Arg
	*MSH6*:exonic:NM_000179.3	NA	Missense	c.2347T>A	p.Cys783Ser
	*MSH6*:exonic:NM_000179.3	NA	Missense	c.3489A>C	p.Glu1163Asp
	*PMS2*:exonic:NM_000535.7	0.0	Missense	c.924G>C	p.Glu308Asp
	*PMS2*:exonic:NM_000535.6	NA	Missense	c.1555T>C	p.Tyr519His
	*PMS2*:exonic:NM_000535.7	0.001	Frameshift	c.2186_2187delTC	p.Leu729GlnfsTer6
**Non-MMR genes**	*APC*:exonic:NM_000038.6	NA	Missense	c.5038C>G	p.Gln1680Glu
	*BMPR1A*:exonic:NM_004329.3	NA	Missense	c.185A>G	p.Tyr62Cys
	*POLE*:exonic:NM_006231.4	NA	Missense	c.73G>A	p.Ala25Thr
	*POLE*:exonic:NM_006231.4	NA	Missense	c.4759G>A	p.Val1587Ile
	*POLE*:exonic:NM_006231.4	NA	Missense	c.4144C>T	p.Arg1382Cys
	*POLE*:exonic:NM_006231.4	NA	Missense	c.3901G>A	p.Val1301Met
	*POLE*:exonic:NM_006231.4	NA	Missense	c.1004T>G	p.Phe335Cys
	*POLE*:exonic:NM_006231.3	NA	Missense	c.4124C>T	p.Ala1375Val
	*POLE*:exonic:NM_006231.3	NA	Missense	c.1337G>A	p.Arg446Gln
	*POLE*:exonic:NM_006231.4	NA	Missense	c.3311C>T	p.Thr1104Met
	*POLE*:exonic:NM_006231.4	NA	Missense	c.3970C>T	p.Arg1324Cys

^a^Minor allele frequency, gnomAD. “NA” = “Not available. ^b^Mismatch repair.

## 4 Discussion

We screened a cohort uniquely consisting of indigenous African patients using an NGS multigene panel for CRC as an advance approach relative to other studies among African continental populations. Twenty-five patients (23.4%) were confirmed to carry a PGV associated with the disease after successful verification (24 PGVs in eight different genes: *APC, BMPR1A*, *MLH1*, *MSH2*, *MUTYH*, *PMS2*, *POLD1*, and *TP53*), yielding a higher rate of PGVs than the average rate reported in the literature (54–57). These previous studies with similar approach to ours also made use of an NGS multigene panel in an early-onset CRC cohort. To our knowledge, on one hand, the lowest rate of PGVs was 10.1%, using a 32-gene panel (55). On the other hand, the highest rate of PGV identification previously reported was 24.5%, using an 18-gene panel (56). However, these were both in a CRC cohort from a Danish population/ethnic group. It is evident from these two studies that (i) the size of the gene panel for CRC testing does not necessarily correlate with the PGVs’ detection rate; (ii) low penetrance genes may influence a low detection rate of PGVs associated with hereditary CRC; and (iii) the cutoff eAOO for inclusion in their cohort may be a factor for a high or low detection rate. Compared to these studies, the detection rate of PGVs in our population-specific CRC multigene panel in a unique and understudied population group, indigenous Africans, was above the expected threshold. Altogether, these support that our multigene panel comprising 14 genes in a unique indigenous African cohort was ample and yielded in a high rate of PGVs associated with CRC.

More than half of the PGVs identified in our cohort would have been missed using traditional methodologies without the NGS multigene panel in patients admitted to the hospital in the last three decades. Inclusion of eAOO younger than 60 years was found to be an acceptable threshold in our cohort of indigenous African patients that helped us include the unresolved cases in our three-decade records. Selection criteria in the NGS multigene panel studies are generally based on LS-associated features such as family history or Amsterdam/revised Bethesda II criteria, which we did not strictly account for when selecting our cohort (58–60). Our cohort, to some extent, was unselected for most of the LS features except for onset age; we included any unresolved indigenous African patients with an eAOO <60 years but not <50 years in our CRC repository. The study by Perkins et al. exemplifies the advantage of an unselected cohort resolving those with inaccurate clinical information recorded who were possibly admitted to the hospital much later than the actual disease onset (58). In our study, patients with an onset of earlier or later than 50 years of age did not show any significant difference for their mutation positivity (**Table 2**). This was best supported by two of our patients whose disease onset was ≥50 years old but presented with a pathogenic, LS-associated MMR variant (**Table 3**), which could be due to limited access to healthcare resulting in late diagnosis. This study thus adds unique evidence to initiate a genetic testing strategy for this population group affected by CRC, and to gauge the potential implications on value of clinical surveillance services and early intervention.

The existing literature stresses the need for more advanced genetic screening studies in indigenous Africans, to which our work contributed (61, 62). Our findings helped us confirm the feasibility of our current NGS panel, which could be interrogated as part of a comprehensive strategy for a systematic CRC genetic screening in South Africa. Our data have provided additional insight into and understanding of the hereditary nature of early-onset CRC in indigenous African patients. The high rate of actionable disease-causing variants in a unique indigenous African cohort of early-onset CRC demonstrates the need for extended genetic screening programs and research in African populations.

The prevalence of LS among study cohorts of different CRC populations varies below 10% (63–65). Our cohort’s prevalence of LS was 13% (14 out of 107), which resolved the genetic diagnosis for our suspected LS patients. However, two of these patients (patient 16 and patient 18) presented with non-classical LS feature of having a cancer diagnosis after the age of 50 years old. It has been reported that indigenous Africans present with CRC at a young age especially in cases where they are found to carry a PGV associated with LS, and we therefore believe that these two LS patients’ relatively older age at cancer diagnosis could be due to either underdiagnosis or, equally as important, other genomic variations in cancer including epigenetic modifiers in this study population (12, 14, 66). Interestingly, only five of the LS patients had a loss of MMR genes reported previously by IHC staining in our laboratories. Without conducting this study, 12 LS patients would have been missed due to lack of or inaccurate IHC testing outcome or insufficient pathological information. This may be due to the unique population group, which may present differently for LS classical features compared to LS in other studied populations such as Europeans and Asians (8, 61, 67, 68). These findings suggest that there may be differences in how LS presents in the same or different populations, and that accurate personalized genetic testing is essential for proper diagnosis. Further research is needed to better understand these differences and improve diagnosis and treatment for LS across all populations.

Identification of novel variants is crucial in terms of identifying founder mutations in especially understudied populations and was expected due to the nature of our cohort being population-specific to indigenous Africans. The NGS 14-gene panel screening consisted of not only novel MMR variants, but also a high proportion of novel non-MMR germline variants. Over 90% of the other PGVs within this cohort were reported only in other population groups including Asian and European sources and not in an African population. Our findings give strong evidence that an advanced genetic screening program in indigenous Africans is a necessity and there is yet much more to explore to provide appropriate personalized treatment and intervention to these understudied indigenous African patients of CRC in South Africa.

Interestingly, as an example of the novel variants, a splice site acceptor pathogenic variant, *MLH1*:c.117-1G>A, was present in two patients (patient 1 and patient 3, **Table 3**), with a strong evidence on pathogenicity: with the position being well conserved, and the “Human Splicing Finder” predicting a functional effect on splicing (**Supplementary Figure 3**) (69). A different variant at this splice site has been reported in a Spanish family and has been proven to disrupt RNA splicing, confirmed with segregation analysis among the CRC-affected family members (70). At the time of the study, the variant at the same position c.117G>T was a VUS but present in the affected family members. RNA analysis by Ruiz et al. confirmed that the variant is disease-causing (70). Further screening of the novel variants in wider CRC patient cohorts will help determine whether the changes we have detected are founders in the indigenous Southern African population.

It is worth noting that identification and verification of *PMS2* variants in CRC NGS multigene panel studies are challenging and require extra effort (36, 71, 72). Most of the studies of NGS panel testing do not include specific methodology for *PMS2* variants to avoid pseudogene interference (5, 57). Our study involved a specific technique for these variants, and we were able to show whether the *PMS2* variants were gene-specific, which gave strength in the verification process. Patients who presented with a *PMS2* PGV and verified by LR-PCR were resolved. In the patients where the gene-specific LR-PCR showed that the variants were absent were marked as unresolved in their molecular diagnosis of CRC in our database.

Our NGS analysis observed some unexpected but critical findings. Of these, one was the pathogenic *MLH1:c*.1731G>A variant identified in patient 20, which was initially filtered out of the analyses as it was categorized as a synonymous variant (by the Ion reporter annotation). However, during a second manual inspection, it was found to be a splice acceptor site variant. This variant has been reported in the literature in multiple affected CRC individuals in unrelated studies, albeit of European (e.g., Germany, Italy, France, and Poland) ancestry (73–76). Identification of this variant highlights the importance of the annotation and filtering procedure and may suggest that an optimized strategy for annotating splice sites variants more accurately is necessary. Secondly, after variant verification, patient 21 was found to carry two pathogenic MMR variants: *MSH2*:c.1923T>A (novel) and *PMS2*:c.2192_2196delTAACT. One of the studies that reported this variant also performed a gene-specific verification technique to avoid pseudogene interference and was able to show the presence of the variant in the actual gene (38). However, our verification by LR-PCR showed that the variant was absent in the actual *PMS2* gene. This is consistent with this patient only having one LS mutation in *MSH2*, which is a more likely/expected event. Importantly, Chong et al. reported an “African” *PMS2* pathogenic variant to be changed to a *PMS2CL* variant, making it a “pseudo” variant (77). Our *PMS2* variants found in this study that failed verification for the presence of the variant in the actual gene are likely to be pseudogene variants rather than a PGV associated with CRC in this population. Additional work is undertaken to finalize these findings.

Another unexpected finding was a non-MMR, but novel pathogenic variant, *POLD1*:c.1265_1266insG found in patient 14 (eAOO <30 years). This finding gives support to the previous published studies that variants in the proofreading activity domain genes may be included in testing for familial CRC, as candidate variants are being identified in affected individuals (29–31). It serves as a good example of novel *POLD1* pathogenic variants associated with hereditary CRC and may be a population-specific candidate variant.

The number of VUSs identified in our study was higher than expected in the general population, yet is expected in such understudied populations as the indigenous African population in South Africa due to lack of data reported for this population group (18, 78). We also found a high proportion of VUSs in the MMR genes (47%) among the VUSs that suggest pathogenicity at some level, together with changes in other genes, e.g., *POLE*, *APC*, and *BMPR1A*. These variants could be better classified if further testing was available such as tumor sequencing. For instance, patient 24 carries a single PGV in *MUTYH* and, thus, has a small increased risk for CRC, which is known to lead to late cancer progression (23, 79). However, the patient was already diagnosed with CRC at an early age, which progressed in a short time, spreading to the liver. It is worth noting that this patient carries three VUSs in *POLE*. One of these variants, as an example, *POLE*:c.3901G>A, was evaluated, and it could have an effect on pathogenicity when combined with the heterozygous *MUTYH* variant. Based on the ACMG criteria, *POLE*:c.3901G>A is classified as likely pathogenic when it is present with another heterozygous PGV. To confirm this, further investigation and additional information especially involving patient’s tumor sequencing would be necessary.

Our study had some limitations. We had difficulties in finding available biological material for DNA extraction for the samples that were either depleted, failed QC, or missing. This led to the exclusion of those patients from the NGS panel screening, and we may have missed resolving these probands and their families. Also, we were only able to include the tumor localization and sidedness but no other pathological information or family history that would help us clearly classify each patient with specific types of hereditary CRC. For instance, only three of the mutation-positive patients in our cohort had a family history (**Table 3**). Additionally, there may be VUSs unique to the population group in the study to be reclassified as PGV, which require additional evidence and additional studies before their reclassification could possibly be made. The panel testing produced no PGVs, but three VUSs in *MSH6* were carried by five patients (4.7%); this, however, is in line with reports that *MSH6* variants are less prevalent (54). Owing to the insufficient number of studies in African populations, a high percentage of VUSs is reported in literature and is a current drawback in the field (78). Our work adds value to the literature to support that a high proportion of VUSs are often observed in African populations and extensive studies are required to overcome this uncertainty.

Our study suggests some critical future work to overcome the limitations and resolve the remaining cases yet with an unknown molecular diagnosis, which are already in place in our laboratory. These include as follows:

(i) An expanded variant calling and annotation tool is being implemented to improve the pickup rate of PGVs.
(ii) The classification of the VUSs could be explored, especially for patients in whom we did not find any germline findings as expected based on previous testing results (e.g., IHC).
(iii) Those with a very strong clinical background suggesting inheritance yet with no PGV after the NGS screening are being analyzed for copy number variants and methylation analysis and will then undergo whole exome sequencing (WES). Families in which DNA samples (or blood/saliva) of at least two family members (one from each generation) are available will be prioritized for WES.

In conclusion, our study demonstrates that a genetic screening program is an urgent necessity in an African setting for affected CRC patients. In our cohort, almost one in four patients (1:4) had an actionable disease-causing variant and a high percentage of the patients carried a novel PGV that could only be detected by a CRC NGS 14-multigene panel analysis. This study supports the evidence for our hypothesis that investigating the germline variants in affected indigenous African individuals with early-onset CRC can provide more insights and inferential information on the genetic nature of CRC in this population. As part of our ongoing patient support program to translate research into diagnostics, our clinical team will convey these test results to the patients for possible discussion on the intervention and possibilities for cascade testing for their family members. This work adds to the literature and supports the overdue genetic research in indigenous African patients.

## Data availability statement

The original contributions presented in the study are publicly available. This data can be found here: https://www.ncbi.nlm.nih.gov/bioproject/PRJNA1005297. Accession number: PRJNA1005297.

## Ethics statement

The studies involving humans were approved by The Human Research Ethics Committee (HREC), Faculty of Health Sciences, University of Cape Town (UCT). The studies were conducted in accordance with the local legislation and institutional requirements. The participants provided their written informed consent to participate in this study.

## Author contributions

SY: Conceptualization, Data curation, Formal Analysis, Investigation, Methodology, Validation, Visualization, Writing – original draft, Writing – review & editing. TM: Data curation, Formal Analysis, Methodology, Writing – review & editing. UA: Data curation, Methodology, Resources, Writing – review & editing. RC: Conceptualization, Investigation, Supervision, Writing – review & editing. GR: Conceptualization, Data curation, Investigation, Methodology, Project administration, Supervision, Writing – review & editing. PG: Data curation, Resources, Writing – review & editing. RR: Conceptualization, Formal Analysis, Funding acquisition, Investigation, Project administration, Resources, Supervision, Validation, Writing – review & editing.

## Footnotes

1
https://www.nicd.ac.za/centres/national-cancer-registry/

2
https://ionreporter.thermofisher.com/ir/

3
https://www.ncbi.nlm.nih.gov/clinvar/

4
https://www.ncbi.nlm.nih.gov/snp/

5
https://franklin.genoox.com/

6
https://www.telethon.jp.
