# Supplementary material for: Genetic insights: High germline variant rate in an indigenous African cohort with early-onset colorectal cancer
Source: Front Oncol. 2023 Oct 27;13:1253867. doi: 10.3389/fonc.2023.1253867 (PMC10642181; doi:10.3389/fonc.2023.1253867)
Supplement: Supplementary file 1 [file Image_1.pdf]

Supplementary Material

Genetic insights: High germline variant rate in an indigenous African cohort with early-onset colorectal cancer

Safiye Yildiz, Takudzwa N. Musarurwa, Ursula Algar, Ramadhani Chambuso, George Rebello, Paul A. Goldberg, and Raj Ramesar*.

* Correspondence: Raj Ramesar: raj.ramesar@uct.ac.za

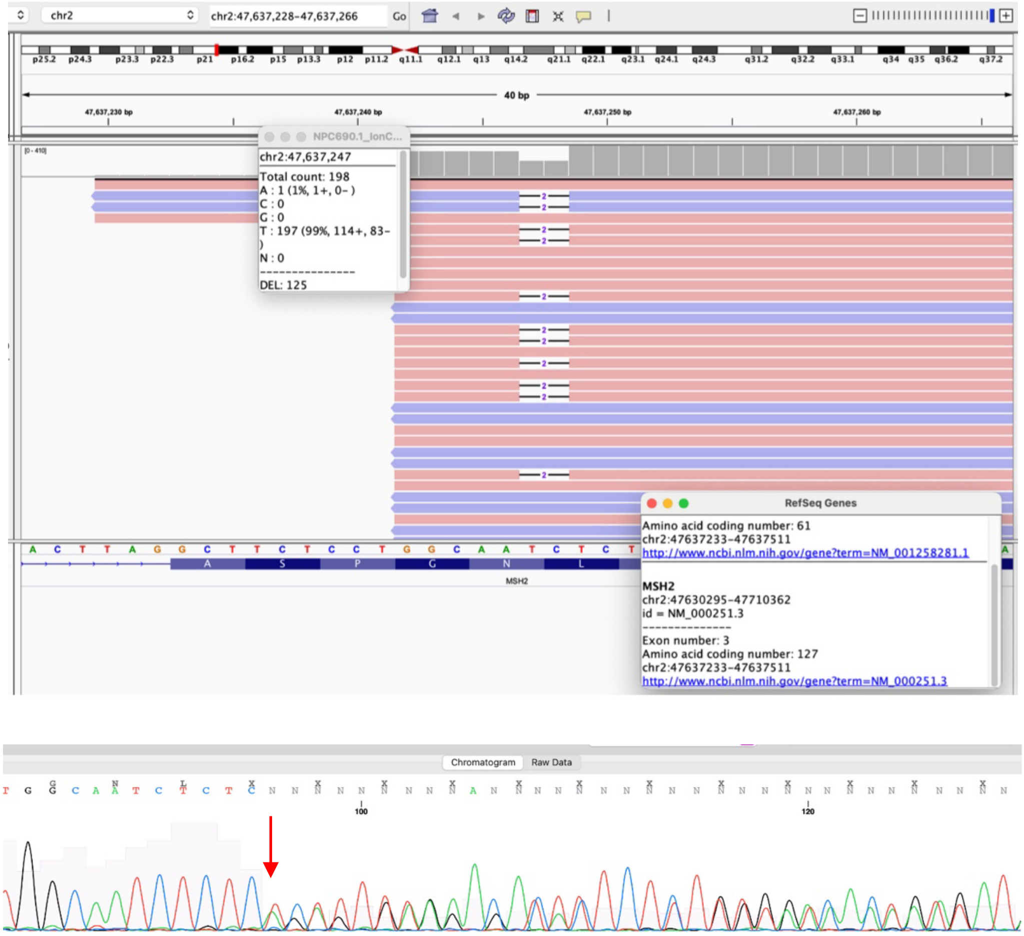

Supplementary Figure 1. Evidence for the two base-pair deletion in *MSH2* in patient 17, confirmed after manual inspection using the Integrative Genomics Viewer software (top). The presence of the variant *MSH2*:c.387_388delTC was then verified in patient 17 by Sanger sequencing (bottom).
