# Supplementary material for: Genetic insights: High germline variant rate in an indigenous African cohort with early-onset colorectal cancer
Source: Front Oncol. 2023 Oct 27;13:1253867. doi: 10.3389/fonc.2023.1253867 (PMC10642181; doi:10.3389/fonc.2023.1253867)
Supplement: Supplementary file 2 [file Image_2.pdf]

Supplementary Material

Genetic insights: High germline variant rate in an indigenous African cohort with early-onset colorectal cancer

Safiye Yildiz, Takudzwa N. Musarurwa, Ursula Algar, Ramadhani Chambuso, George Rebello, Paul A. Goldberg, and Raj Ramesar*.

* Correspondence: Raj Ramesar: raj.ramesar@uct.ac.za

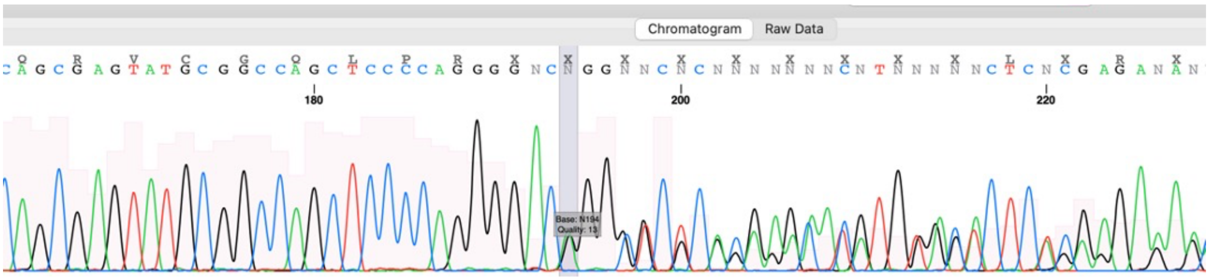

Supplementary Figure 2. Verification of the *PMS2* variant, c.1579_1580delAG in patient 18 using long-range PCR followed by Sanger sequencing.
