# Supplementary material for: Genetic insights: High germline variant rate in an indigenous African cohort with early-onset colorectal cancer
Source: Front Oncol. 2023 Oct 27;13:1253867. doi: 10.3389/fonc.2023.1253867 (PMC10642181; doi:10.3389/fonc.2023.1253867)
Supplement: Supplementary file 3 [file Image_3.pdf]

Supplementary Material

Genetic insights: High germline variant rate in an indigenous African cohort with early-onset colorectal cancer

Safiye Yildiz, Takudzwa N. Musarurwa, Ursula Algar, Ramadhani Chambuso, George Rebello, Paul A. Goldberg, and Raj Ramesar*.

* Correspondence: Raj Ramesar: raj.ramesar@uct.ac.za

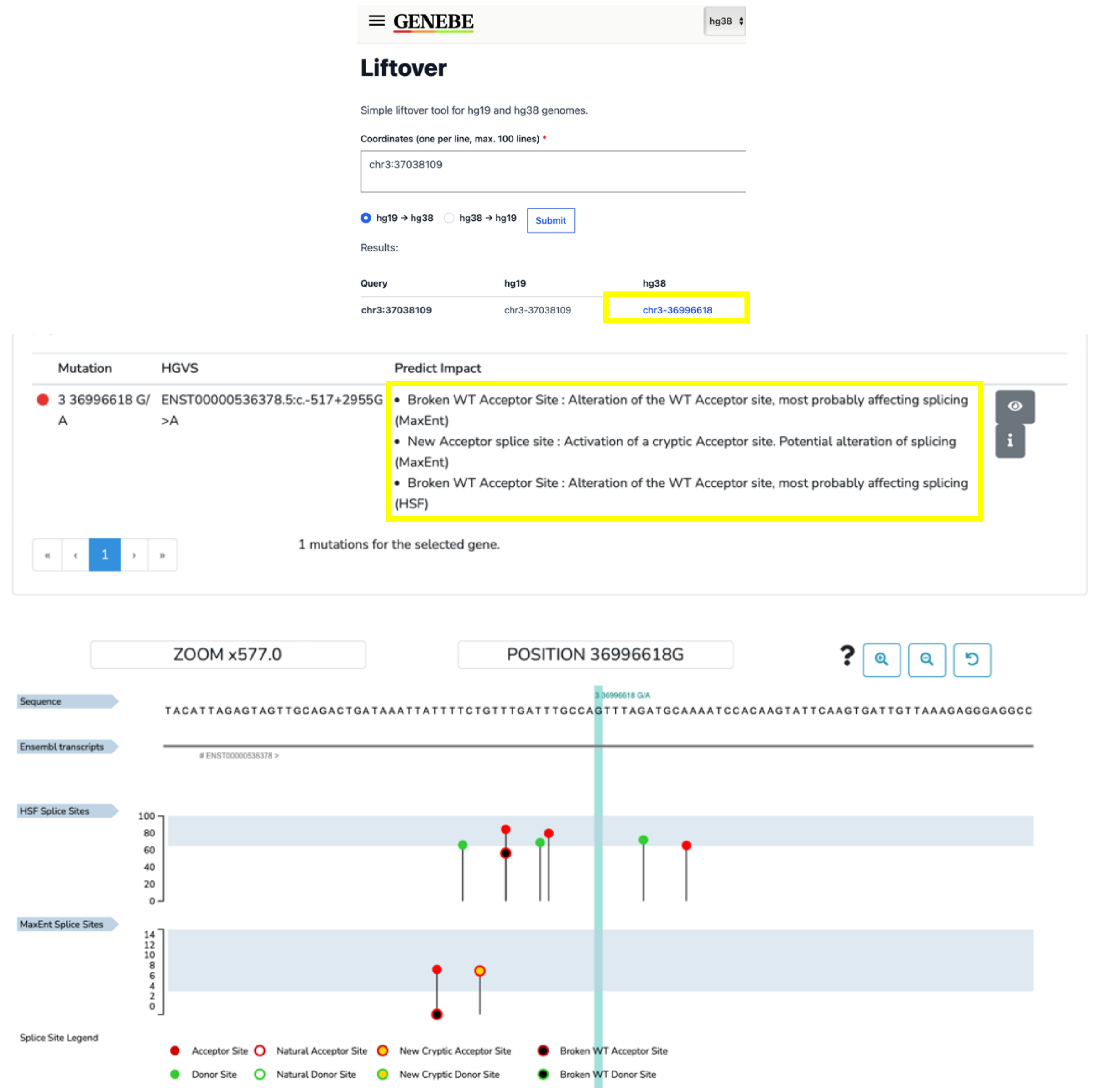

Supplementary Figure 3. Human Splicing Finder (HSF) tool to predict splicing effect was used for the splice site variant, *MLH1*:c.117-1G>A being present in two patients 1 and patient 3. Liftover was first used to convert the original reference genome position of the variant, hg19 to hg38 (top), and then put through the HSF tool for splicing effect prediction; which found potential alteration of splicing (bottom).
